# Redox–Metabolic Crosstalk in Pediatric MASLD: Biomarkers, Mechanisms, and the Emerging Role of Selenium

**DOI:** 10.3390/ijms27177575

**Published:** 2026-08-24

**Authors:** Sanja Panic Zaric, Teofana-Otilia Bizerea-Moga, Rade Vukovic, Maja Janicijevic, Raluca Isac, Tudor Voicu Moga, Alina Popescu

**Affiliations:** 1 Mother and Child Health Care Institute of Serbia “Dr Vukan Cupic”, 11070 Belgrade, Serbia; sanja.pnc@gmail.com (S.P.Z.); radevukovic9@gmail.com (R.V.); cehicmaja95@gmail.com (M.J.); 2 Department of Pediatrics, First Pediatric Clinic, Center for Research on Growth and Developmental Disorders in Children, ‘Victor Babeș’ University of Medicine and Pharmacy Timișoara, Eftimie Murgu Sq no. 2, 300041 Timișoara, Romania; bizerea.teofana@umft.ro; 3 Emergency Clinical Hospital for Children ‘Louis Țurcanu’, Iosif Nemoianu 2, 300011 Timișoara, Romania; 4 School of Medicine, University of Belgrade, 11000 Belgrade, Serbia; 5 Department of Pediatrics—Third Pediatric Clinic, ‘Victor Babeș’ University of Medicine and Pharmacy Timișoara, Eftimie Murgu Sq no. 2, 300041 Timișoara, Romania; 6 Department of Internal Medicine—Gastroenterology Clinic, Advanced Regional Research Center in Gas-Troenterology and Hepatology, ‘Victor Babeș’ University of Medicine and Pharmacy Timișoara, Eftimie Murgu Sq no. 2, 300041 Timișoara, Romania; moga.tudor@umft.ro (T.V.M.); popescu.alina@umft.ro (A.P.); 7 Gastroenterology and Hepatology Clinic, ‘Pius Brînzeu’ County Emergency Clinical Hospital, Liviu Rebreanu 156, 300723 Timișoara, Romania

**Keywords:** MASLD, oxidative stress, selenium

## Abstract

Childhood obesity is an important global health issue closely related to metabolic syndrome and metabolic dysfunction-associated fatty liver disease (MASLD), currently the most common chronic liver disease in children. Oxidative stress and insulin resistance are the driving forces in hepatocyte injury and disease progression. There is growing interest in new biomarkers for detecting and monitoring metabolic and hepatic changes. We aimed to synthesize current evidence regarding biomarkers of oxidative stress and MASLD, and the potential modulatory role of selenium in pediatric populations. A literature search of PubMed, Scopus, Web of Science, and the Cochrane Library identified studies in children and adolescents (0–18 years) published between January 2000 and December 2025. Associative diagnostic biomarkers included indicators of oxidative damage, antioxidant-defense markers, and integrated oxidative stress indices, which appear promising but lack pediatric reference ranges, while prognostic biomarkers capture the inflammatory consequences of redox imbalance but with limited liver specificity. Metabolomics profiling and multiomics approaches remain experimental. Selenium, an essential trace element with antioxidant properties, plays a role in maintaining redox balance through selenoproteins, but pediatric evidence is limited and at times contradictory. Few biomarkers are clinically ready. Further longitudinal studies in pediatric populations are needed to better clarify the role of selenium, as well as to support the development of age-appropriate diagnostic strategies utilizing multiomics for MASLD, empowering clinicians to implement timely interventions in childhood obesity.

## 1. Introduction

The pandemic of pediatric obesity is one of the main global public health issues, accompanied by a rise in the incidence of metabolic syndrome (MetS) in the pediatric population, leading to an elevated risk of cardiometabolic consequences later in life [1]. Metabolic dysfunction-associated fatty liver disease (MASLD) is the main hepatic manifestation of MetS, with the potential to progress to cirrhosis silently, emphasizing the importance of early detection [2].

Fatty liver disease nomenclature has evolved substantially. Nonalcoholic fatty liver disease (NAFLD) and nonalcoholic steatohepatitis (NASH) were historically defined by excluding significant alcohol exposure and secondary causes. The metabolic dysfunction-associated fatty liver disease (MAFLD) framework subsequently proposed a positive diagnosis based on hepatic steatosis plus overweight/obesity, type 2 diabetes, or metabolic dysregulation, thereby emphasizing metabolic drivers [3]. In 2023, a multisociety Delphi consensus endorsed by United European Gastroenterology introduced steatotic liver disease (SLD) as the overarching category and metabolic dysfunction-associated steatotic liver disease (MASLD) for steatosis accompanied by at least one cardiometabolic risk factor, and metabolic dysfunction-associated steatohepatitis (MASH) replaced NASH [4]. ESPGHAN and other pediatric liver societies endorsed the new nomenclature while emphasizing pediatric-specific features and age-appropriate assessment [5], as reflected in the updated ESPGHAN position paper on pediatric MASLD [6]. Accordingly, to ensure clarity and terminological consistency, MASLD and MASH are used throughout the narrative synthesis, irrespective of the terminology employed in the original study [3,4,5,6].

The mutual underlying problem of obesity and MASLD is a combination of low-grade inflammation and insulin resistance (IR), resulting in increased oxidative stress (OS), further promoting liver injury via mitochondrial dysfunction, increased production of reactive oxygen species (ROS), lipid peroxidation, and reduction of endogenous antioxidant defense [7]. IR both promotes OS and may itself be exacerbated by it, leading to a vicious cycle [8]. In this context, OS has been proposed as a central mechanism connecting childhood obesity with the development of MASLD [9].

The traditional biomarkers alanine aminotransferase, aspartate aminotransferase, and gamma-glutamyltransferase have been extensively used as indirect indicators of hepatic injury and metabolic dysfunction [1]. Unfortunately, they all have very limited sensitivity, variable cutoffs and a low predictive value. During recent years, novel inflammatory, immune and OS biomarkers of metabolic alterations in MASLD have been the focus of research, alongside the evaluation of antioxidant defense through redox modulation, where selenium has a significant role as a part of selenoproteins and enzymes like glutathione peroxidase (GPx).

Although many studies have examined individual biomarkers, pathophysiological mechanisms and components of MASLD, a comprehensive overview analysis of the relationship between OS and MASLD, as well as the role of selenium in pediatric populations, has not yet been performed. Keeping in mind that the data regarding children are still controversial, the aim of this review was to provide a structured and critical synthesis of known evidence regarding biomarkers of OS and MASLD classified by their diagnostic and clinical utility, as well as to analyze the available evidence on selenium and selenoproteins in this context, while highlighting research gaps and future directions.

## 2. Literature Search Strategy

### 2.1. Study Design

This review was designed as a narrative review rather than a systematic review due to heterogeneity in study designs, outcome definitions, and pediatric age-group classifications across the literature.

### 2.2. Search Strategy and Databases

A structured literature search was performed in February 2026 using PubMed, Scopus, Web of Science, and the Cochrane Library and included studies published from January 2000 to December 2025. The search included different combinations of the following terms: “oxidative stress”, “children”, “pediatric”, “adolescent”, “NAFLD”, “NASH”, “MAFLD”, “MASLD”, “MASH”, “steatotic liver disease”, “fatty liver”, “selenium”, and “biomarkers”.

### 2.3. Inclusion and Exclusion Criteria

The inclusion criteria involved studies regarding pediatric populations aged 0–18 years examining OS markers, inflammatory biomarkers, adipokines, or selenium/selenoprotein-related markers in relation to obesity, MetS, or MASLD, including original research articles, systematic reviews, and meta-analyses published in English.

The exclusion criteria involved studies with adults, non-English publications without available translations, case reports, editorials, and conference abstracts, and studies conducted before 2000 except for landmark studies.

### 2.4. Study Selection and Data Extraction

**Figure 1:**
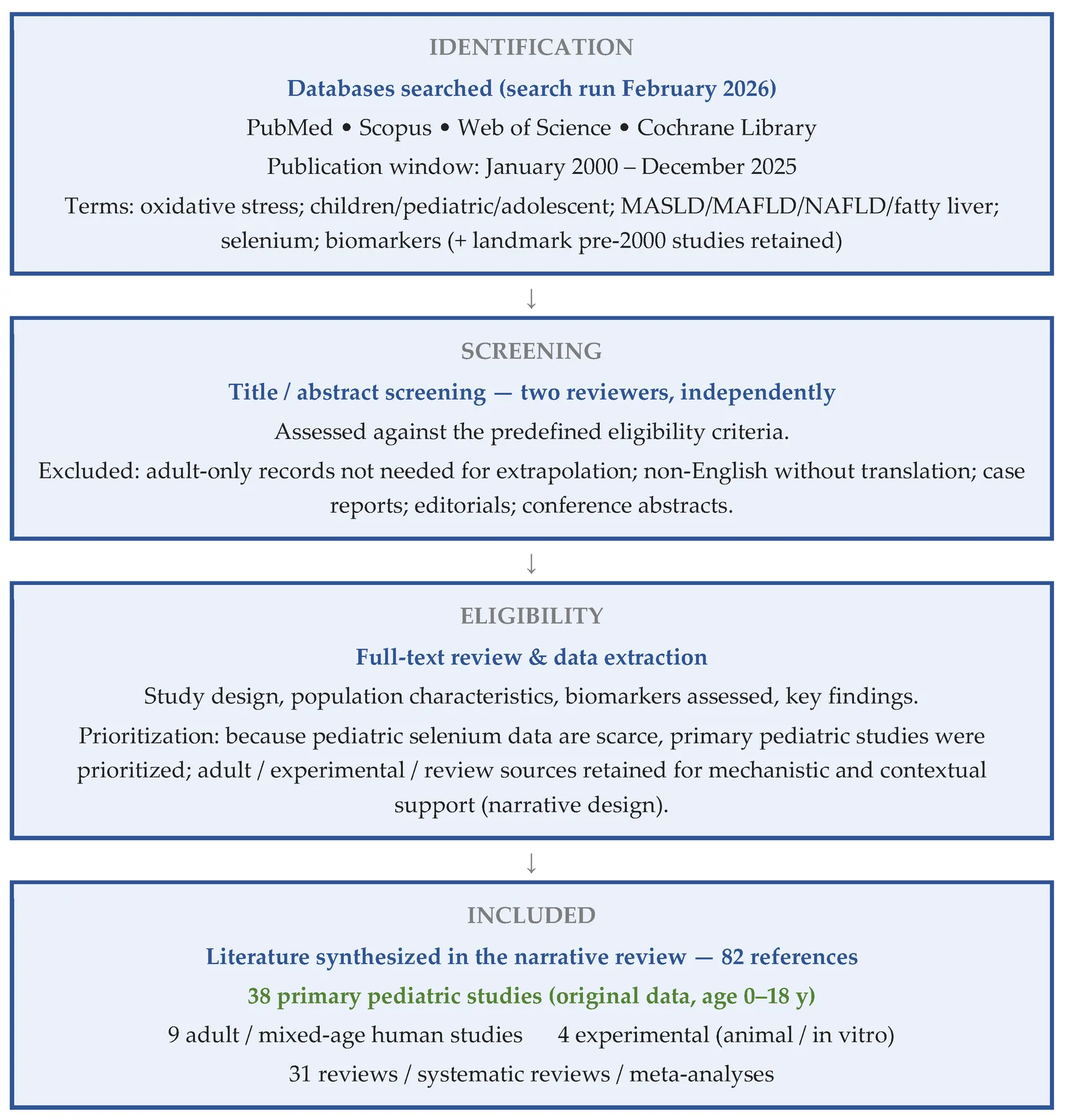
Literature selection and prioritization for the narrative review.

Initial screening of titles and abstracts was performed by two members of the research team independently, followed by full-text review of potentially eligible studies. Data extracted included study design, population characteristics, biomarkers assessed and key findings. To improve methodological transparency, Figure 1 provides a flowchart illustrating the literature identification, screening, eligibility assessment, and final selection of studies included in the review.

### 2.5. Bias and Quality Considerations

A formal risk-of-bias tool was not applied, consistent with the narrative review format. However, the strengths and limitations of individual studies are discussed throughout the text.

## 3. Literature Review

### 3.1. The Role of OS in the Pathophysiology of MASLD

The complex pathophysiology of MASLD revolves around three intertwined processes paving the road from obesity to liver injury. The first process is centered around lipotoxicity because of fatty acids released from dysfunctional and insulin-resistant adipocytes, caused by the accumulation of triglyceride-derived toxic metabolites in ectopic tissues (liver, muscle and pancreatic beta cells), following induction of inflammatory mechanisms, cellular dysfunction, and lipoapoptosis. Complex communication between dysfunctional adipocytes and the liver mediated by macrophages and other immune cells further propagates the development of lipotoxic liver disease [10]. The second process is the increase in OS due to increased production of ROS, which promotes inflammation and cytotoxicity, therefore leading to metabolic dysfunction-associated steatohepatitis (MASH) and fibrosis. The third process is further development of hepatocyte dysfunction due to excessive OS and reduced replication of mature hepatocytes supporting progenitor cell expansion, potentially resulting in hepatocyte death, cirrhosis and hepatocellular carcinoma [11].

Beyond adipose–liver and gut–liver crosstalk, an emerging spleen–liver axis may contribute to the immunometabolic amplification of MASLD. Through portal circulation, splenic cytokines and immune-cell populations may influence hepatic inflammation and fibrogenesis, suggesting that the spleen could function as an active immunological participant rather than solely as a passive marker of portal hypertension. Experimental obesity models have demonstrated coordinated changes in splenic and hepatic immune-cell populations, including enrichment of myeloid-derived suppressor cells and natural killer T cells associated with fatty liver inflammation. This mechanism may be particularly relevant to pediatric MASLD, which can exhibit a portal-predominant histological pattern characterized by portal inflammation and portal/periportal fibrosis. In children with biopsy-confirmed MASLD, portal inflammation has been independently associated with hepatic fibrosis and features of metabolic syndrome. Nevertheless, direct pediatric evidence demonstrating a causal spleen–liver mechanism remains limited and warrants prospective investigation [12,13,14,15,16].

Different factors such as pollution, a suboptimal diet, smoking, a sedentary lifestyle, and stress have an impact on promoting OS, which could lead to early-onset aging and the evolution of different conditions, for example cardiovascular, neurological and inflammatory diseases, IR, and MetS [17]. OS is the result of an imbalance between the production of ROS or reactive nitrogen species (RNS) and the antioxidant defenses which support deoxyribonucleic acid (DNA) impairment and cell damage and could lead to cell death [18]. ROS and RNS may lead to inflammation of the intestinal mucosa, which influences the absorption of nutrients and consequently triggers a generalized inflammatory response [17]. MASLD is characterized by liver injury, which is the result of the prevailing force of OS despite antioxidant defenses needed for safeguarding cellular integrity. Moreover, OS promotes MASLD progression by initiating inflammation and fibrosis [8]. The principal generators of ROS are Kupffer cells, which play a role in inflammatory reactions by producing different cytokines, adhesion molecules and chemokines. Altered hepatocytes enhance paracrine activity, inflammation and fibrosis, and the whole transformation leads to advancement of fibrosis and consequently liver cirrhosis [19].

A majority (83%) of children with MASLD have signs of oxidative injury, with evidence of reduced activity of antioxidant enzymes, superoxide dismutase (SOD) and GPx in patients with MASLD [20,21].

**Figure 2:**
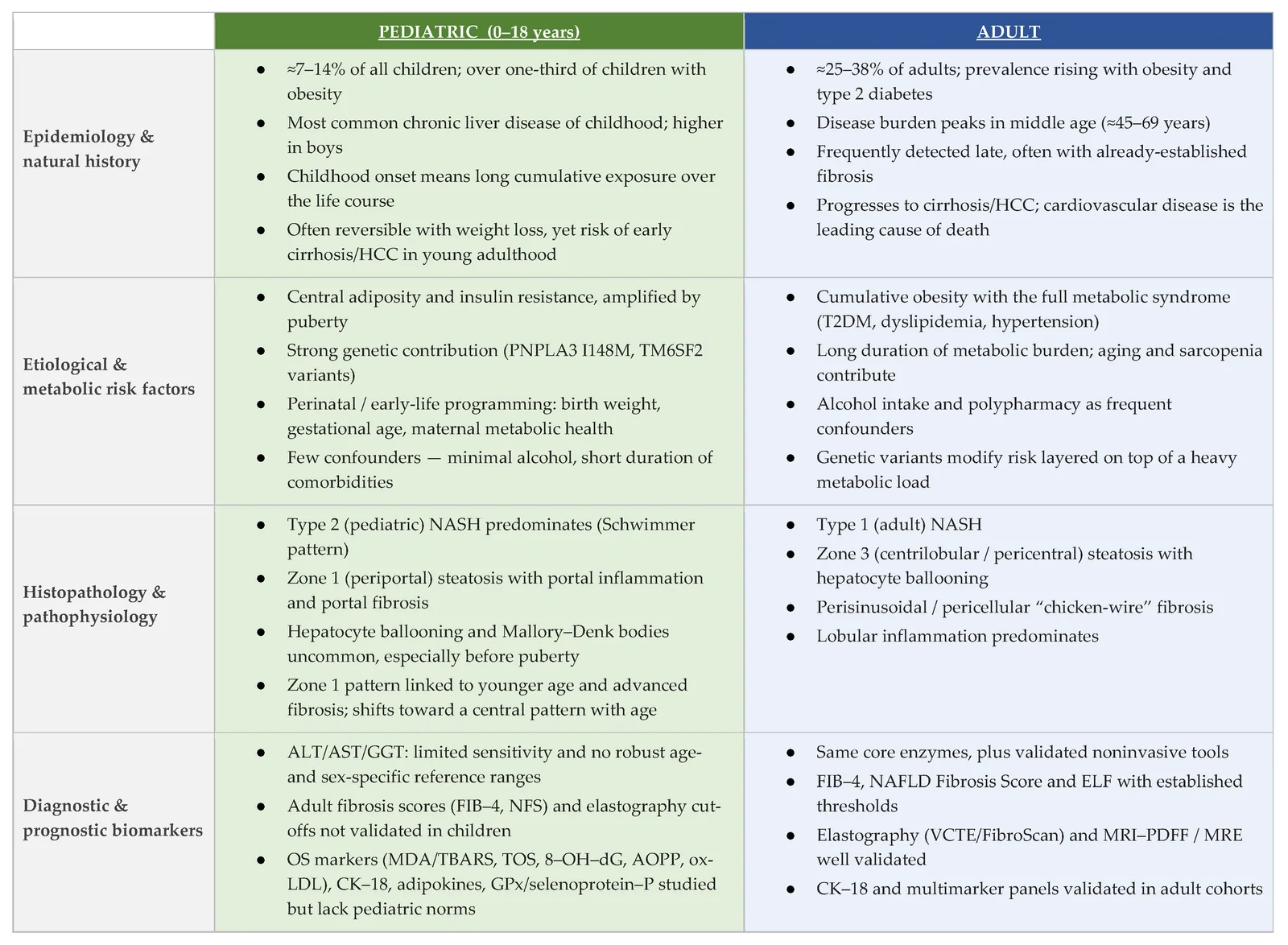
Comparative overview of pediatric and adult MASLD [1,2,7,8,9,10].

The clinical and biological characteristics of MASLD differ between pediatric and adult populations, reflecting differences in epidemiology, metabolic risk factors, disease progression, and underlying pathophysiological mechanisms. Key similarities and differences between pediatric and adult MASLD, including epidemiology and natural history, etiological and metabolic risk factors, histopathology and pathophysiology, and diagnostic and prognostic biomarkers, are summarized in Figure 2.

### 3.2. Associative Diagnostic Biomarkers

Keeping in mind the central role of OS in MASLD, biomarkers that reflect the link between oxidant production and antioxidant defenses may offer value in early diagnostic assessment of MASLD. These diagnostic biomarkers can be divided into indicators of oxidative damage, antioxidant-defense markers, and integrated oxidative stress indices.

#### 3.2.1. Markers of Oxidative Damage (Table 1)

The most frequently measured OS biomarkers in MASLD include nitric oxide, lipid damage products (lipid peroxides, thiobarbituric acid reactive substances (TBARS), malondialdehyde (MDA), total oxidant status (TOS), hydroperoxides, 8-isoprostane, 4-hydroxynonenal, protein oxidation products (protein carbonyl, nitrotyrosine), DNA oxidation product 8-hydroxy-2′-deoxyguanosine (8-OH-dG), and cytochrome P450 2E1 [22,23,24,25]). Also, F2-isoprostanes are considered among the most reliable biomarkers in vivo, due to their chemical stability and sensitivity to changes in OS [26]. In pediatric patients with obesity, elevated levels of TBARS, isoprostane and TOS were detected, alongside a paradoxical increase in total antioxidant status, while levels of antioxidant-defense markers (retinol, β-carotene, and vitamin E) were significantly reduced [27]. Advanced oxidation protein products (AOPPs) were higher in overweight and obese children and adolescents compared to lean controls and even more elevated in patients with MetS [28]. Also, AOPPs showed a positive correlation with central obesity, triglycerides and insulin [28,29]. Obese children had higher MetS prevalence and also higher levels of oxidized low-density lipoproteins (ox-LDLs) compared to normal-weight and overweight children. Also, ox-LDL levels are positively correlated with total cholesterol, triglycerides and LDL-cholesterol [30,31]. Oxidized high-density lipoproteins (ox-HDLs) were higher in the adolescents with MASLD compared to the obese group of adolescents [31].

**Table 1:** Associative diagnostic biomarkers. Markers of oxidative damage (oxidant/damage products).

Biomarker	Mechanism	Clinical Relevance	Limitations	Clinical Readiness
**MDA, TBARS, lipid peroxides** [22,27,32,33]	Lipid-peroxidation end-products; indicators of oxidative membrane damage by ROS.	Elevated in obese children and patients with MASLD.	No standardized pediatric reference ranges; sex-specific differences noted; not liver-specific.	**EMERGING** Detectable in serum; standardization and reference ranges needed.
**F2-isoprostanes, 8-isoprostanes** [26,27,34,35]	Formed by non-enzymatic peroxidation of arachidonic acid; gold-standard in vivo lipid-peroxidation marker.	Elevated in obese children; baseline TAC inversely predicts F2-isoprostane reduction after dietary intervention.	Limited laboratory availability; high cost; pediatric data limited.	**EMERGING** Most reliable OS markers; limited to specialized labs.
**8-OH-dG** [11,20,22]	DNA oxidation product; marker of oxidative DNA damage.	Reflects oxidative DNA damage; weakly correlated with F2-isoprostanes.	Not validated in routine pediatric practice; technically demanding; limited pediatric data.	**RESEARCH ONLY** Research use only; no clinical pediatric validation.
**Protein carbonyls, nitrotyrosine** [11,20,22]	Protein oxidation products reflect cellular oxidative injury.	Reflect cellular oxidative injury; elevated in pediatric MASLD.	Not validated in pediatric practice; technically demanding; limited data.	**RESEARCH ONLY** Research use only; no clinical pediatric validation.
**AOPPs** [28,29]	Dityrosine-containing cross-linked protein products from chronic protein oxidation; complements carbonyls/nitro tyrosine.	Elevated in pediatric obesity and oxidized-lipid-associated protein damage.	Assay not standardized in children; overlaps with inflammation; not liver-specific.	**RESEARCH ONLY** Research use only; pediatric data limited.
**ox-LDL, ox-HDL** [30,31,36]	Oxidatively modified LDL and its receptor; links lipid oxidation to endothelial dysfunction and atherogenesis.	ox-LDL and ox-HDL elevated in obesity/MetS; mediate ROS-driven endothelial injury; cardiometabolic relevance.	Not liver-specific; reflects systemic atherogenic oxidation; pediatric reference ranges lacking.	**EMERGING** Cardiometabolic marker; needs pediatric validation.
**4-Hydroxynonenal (4-HNE)** [22,23]	Reactive aldehyde product of lipid peroxidation; forms protein adducts; pro-fibrogenic stimulus for hepatic stellate cells.	Elevated in MASLD; promotes hepatic fibrosis; lacking pediatric data.	Technically demanding measurement; no standardized pediatric assays.	**RESEARCH ONLY** Mechanistically important; no pediatric clinical assay.
**Hydroperoxides** [22,36]	Early lipid-peroxidation products; precursors to MDA and 4-HNE.	Reflect early-stage oxidative lipid damage; elevated in obese children.	Chemically unstable; rapidly converted to secondary products; no standardized pediatric assays.	**RESEARCH ONLY** Instability limits routine clinical use.
**Nitric oxide (NO)** [22,24]	Free radical and signaling molecule; reacts with superoxide to form peroxynitrite (nitrosative stress).	Elevated in obese children and correlates with OS markers; endothelial NO may be paradoxically reduced.	Very short half-life; measured via stable metabolites (nitrite/nitrate); not liver-specific.	**RESEARCH ONLY** Indirect measurement only; pediatric validation lacking.
**Cytochrome P450 2E1 (CYP2E1)** [22,25]	Microsomal enzyme generating ROS during fatty acid/xenobiotic metabolism; elevated in steatotic liver.	Increased activity in MASLD; contributes directly to hepatic oxidative burden and lipid peroxidation.	Requires liver biopsy or breath tests for direct measurement; not feasible for routine pediatric use.	**RESEARCH ONLY** Liver biopsy required; not feasible for routine use.
**TOS** [8,37,38]	Global oxidant capacity assay measuring total oxidant load in serum; reflects the cumulative oxidative load from all ROS.	Elevated in obese children and adolescents with MASLD; complements TAS as part of integrated OS assessment.	Heterogeneous assay methods; no standardized pediatric reference ranges.	**EMERGING** Measurable in serum; reference ranges lacking in children.

**Legend:** MDA—malondialdehyde, TBARS—thiobarbituric acid reactive substances, ROS—reactive oxygen species, MASLD—metabolic dysfunction-associated steatotic liver disease, TAC—total antioxidant capacity, OS—oxidative stress, 8-OH-dG—8-hydroxy-2′-deoxyguanosine, DNA—deoxyribonucleic acid, AOPPs—advanced oxidation protein products, ox-LDL—oxidized low-density lipoprotein, ox-HDL—oxidized high-density lipoprotein, NO—nitric oxide, 4-HNE—4-Hydroxynonenal, CYP2E1—cytochrome P450 2E1, TOS—total oxidant status. EMERGING—Promising evidence based on pediatric-specific validation, standardized assays, or reference ranges is still lacking. RESEARCH ONLY—Evidence limited to adult/animal studies, single studies, or highly specialized platforms; not feasible for routine clinical use.

#### 3.2.2. Antioxidant-Defense Markers (Table 2)

A cross-sectional study in Mexican children found that obesity was associated with significantly higher catalase (CAT) and GPx enzyme activity compared to normal-weight controls [32]. Furthermore, a significant sex-specific association was observed between obesity and MDA levels and SOD activity, both of which were significantly higher in obese boys [32]. A large cross-sectional study showed a significant positive association between serum total antioxidant capacity (TAC) and TBARS and obesity [33]. These findings suggest a compensatory upregulation of non-enzymatic antioxidant defenses during obesity development in response to oxidative stress. Also, paraoxonase-1 (PON1) is a HDL-attached extracellular esterase which participates in the anti-atherogenic and anti-inflammatory features of HDL. PON1 arylesterase activity is reduced in general and central obesity and hyperinsulinemia and is linked to body mass index (BMI), SOD, CAT, GPx, free thiols, and HOMA-IR [39].

**Table 2:** Associative diagnostic biomarkers. Antioxidant-defense markers.

Biomarker	Mechanism	Clinical Relevance	Limitations	Clinical Readiness
SOD, GPx, CAT [20,22,32]	Antioxidant enzymes: SOD converts harmful superoxide radicals into hydrogen peroxide and oxygen; GPx neutralizes peroxides; CAT converts H2O2 to water.	Altered activity in obesity and MASLD (often compensatory).	Variable results across laboratories; no established pediatric reference ranges; elevated levels may be compensatory.	**EMERGING** Measurable in serum/blood; no standardized pediatric assay.
PON1 arylesterase [39,40]	HDL-associated antioxidant esterase protecting LDL/HDL from oxidation; anti-atherogenic.	Decreased in pediatric obesity, hypertension, hyperinsulinemia; correlates with BMI, CRP, SOD/CAT/GPx, HOMA-IR.	Gender-dependent activity; influenced by HDL/phenotype; no pediatric reference ranges.	**EMERGING** Well studied in pediatric MetS; needs standardization.
TAC [27,33,34,35]	Global antioxidant capacity assay reflects total non-enzymatic antioxidant defense.	Shows biphasic pattern in obesity: initial compensatory increase followed by depletion with progressive metabolic dysfunction; useful for monitoring oxidative balance.	Low specificity; heterogeneous assays; direction varies by disease stage; no standardized pediatric reference ranges.	**EMERGING** Useful for monitoring; low specificity.
Retinol, beta-carotene, vitamin E [27,41]	Fat-soluble antioxidant vitamins scavenging free radicals; non-enzymatic antioxidant reserve.	Reduced in obese children alongside elevated OS markers.	Influenced by dietary intake and fat malabsorption; correlate with plasma lipids; not liver-specific.	**EMERGING** Routinely measurable; not liver-specific; dietary confounding.
sUA/sCr ratio [42]	Uric acid is the end-product of purine metabolism; endogenous antioxidant (pro-oxidant at high levels).	Elevated in obese children; sUA/sCr ratio associated with MASLD risk.	Reflects purine metabolism/renal handling, not integrated redox balance; influenced by hydration, diet.	**EMERGING** Accessible and low-cost; pediatric reference ranges needed.

**Legend:** SOD—superoxide dismutase, GPx—glutathione peroxidase, CAT—catalase, MASLD—metabolic dysfunction-associated steatotic liver disease, PON1—paraoxonase-1, BMI—body mass index, CRP—C-reactive protein, HOMA-IR—Homeostatic Model Assessment of Insulin Resistance, MetS—metabolic syndrome, TAC—total antioxidant capacity, OS—oxidative stress, sUA/sCr—serum uric acid to creatinine ratio, **EMERGING**—Promising evidence based on pediatric-specific validation, standardized assays, or reference ranges is still lacking.

In a study of 55 children with obesity, reactive oxygen metabolite levels were significantly elevated, independent of the presence of MetS, while TAC was reduced in children with obesity compared to controls [34]. Furthermore, in a study of 44 children with obesity, baseline serum TAC has been shown to predict the reduction in F2-isoprostane after 10-week dietary intervention [35]. These findings support the use of these markers as both diagnostic and treatment-monitoring tools.

Elevated levels of uric acid are found in cohorts of obese patients, regardless of the presence of MASLD, as well as a positive correlation between serum uric acid and serum creatinine ratio in patients with MASLD [42]. In a study of 1387 obese children, significant positive association between serum uric acid/serum creatinine ratio and risk factors of MASLD was demonstrated [43]. These findings suggest that serum uric acid/serum creatinine ratio is an accessible and clinically relevant OS-related biomarker.

Although proven to be very useful, in order to implement these biomarkers into clinical practice, standardization of measurement, and age-specific reference ranges, a barrier that applies across all three categories, need to be established.

#### 3.2.3. Integrated Indices and Composite Markers of OS (Table 3)

This group of biomarkers is developed with the purpose of combining an oxidant (or oxidative-damage) component with an antioxidant (or defense) component into a single value. Thereby, composite biomarkers demonstrate the stage of balance between oxidative and reductive processes. This group smooths out fluctuations in individual molecules, providing the comprehensive status of an overall oxidative state. Composite biomarkers are divided into three groups: laboratory ratio/index markers, dietary/lifestyle composite scores (exposure-based) and building-block “capacity” assays.

**Table 3:** Associative diagnostic biomarkers. Integrated/composite oxidative stress indices (combining an oxidant arm with an antioxidant arm).

Biomarker	Mechanism	Clinical Relevance	Limitations	Clinical Readiness
**OSI** [44,45]	Ratio integrating total oxidant and total antioxidant status into one value; the canonical redox-balance index.	Discriminates pediatric MetS/obesity from controls; reported in pediatric fatty liver; low-cost, automatable.	Method-dependent; no standardized pediatric reference ranges.	**EMERGING** Most implementable composite; components already in use.
**Thiol/disulfide homeostasis (native thiol, total thiol, disulfide)** [46,47]	Dynamic thiol–disulfide balance via automated Erel–Neşelioğlu method; integrates oxidant/antioxidant thiol pools.	Shifts toward disulfide in pediatric obesity/fatty liver; inexpensive and automatable.	Few pediatric reference ranges; preanalytical sensitivity.	**EMERGING** Inexpensive; growing pediatric use.
**OXY-SCORE; BAP/d-ROMs ratio** [34,48]	Computed composites combining oxidative-damage and antioxidant-potential measures (ROM interpreted against BAP).	Provide global OS estimates; d-ROMs/BAP used in pediatric obesity research.	No established pediatric reference ranges; assay availability limited.	**RESEARCH ONLY** Composite estimates; pediatric data sparse.
**OBS/CDAI (dietary–lifestyle composites)** [49]	Scores integrating dietary/lifestyle pro- and antioxidant exposures.	Associated with MetS/MASLD risk in population studies; link diet–antioxidant exposure to redox status.	Exposure indices, not blood assays; depend on dietary recall; pediatric validation limited.	**RESEARCH ONLY** Exposure composite; conceptual bridge to selenium.

**Legend:** OSI—oxidative stress index, MetS—metabolic syndrome, BAP—biological antioxidant potential, d-ROMs—reactive oxygen metabolites, OS—oxidative stress, OBS—oxidative balance score, CDAI—composite dietary antioxidant potential, MASLD—Metabolic dysfunction-associated fatty liver disease, EMERGING—Promising evidence based on pediatric-specific validation, standardized assays, or reference ranges is still lacking. RESEARCH ONLY—Evidence limited to adult/animal studies, single studies, or highly specialized platforms; not feasible for routine clinical use.

One of the most used is the oxidative stress index (OSI), calculated as the ratio of TOS to total antioxidant status (TAS) (OSI = TOS/TAS × 100) [44]. The main advantages of OSI are that it is very useful for analyzing metabolic risk and monitoring responses to antioxidant therapies, cheap, and automatable [45]. In pediatric populations, TOS, TAS, and OSI were elevated in children with MetS and obesity compared with healthy controls [37,40]. Also, the fasting insulin level and IR were significantly high in the MetS and obese groups and the TOS value in the MetS group was higher than those in both the control and obese groups in a pediatric population [40].

OXY-SCORE is the oxidative part of the test, and it is the measurement of total hydroperoxides by derivatives of reactive oxygen metabolites (dROMs); the antioxidant part is called the OXY Adsorbent Test, which assesses the antioxidant ability of each sample in contesting oxidant action, as well as showing oxidative balance. Daily use of the OXY-SCORE could be challenging because of calculation difficulties, and it has only been utilized with serum as a biological sample [48]. Another calculated composite is the modified ratio, which uses dROMs and biological antioxidant potential (BAP) [50]. The BAP/d-ROM ratio correlated inversely with BMI SDS, supporting obesity as already a driver of systemic oxidative stress in pediatric populations [34].

Additional single-assay indices involve thiol/disulfide homeostasis (native thiol, total thiol, and disulfide measured by the automated Erel–Neşelioğlu method), which is impaired in obese children [46,47].

Dietary–lifestyle composites include the oxidative balance score (OBS) and the Composite Dietary Antioxidant Index (CDAI), which are tools for rating the influence of diet and lifestyle on OS. OBS serves as a marker of OS which evaluates the oxidative balance of a lifestyle using the intake of antioxidants and pro-oxidants. Dietary OBS involves different pro- and antioxidants, among them selenium. The CDAI shows total dietary antioxidant intake, which includes six dietary antioxidants, namely vitamins A, C, and E, carotenoids, selenium, and zinc [49].

As with the damage and antioxidant-defense markers, the principal barrier to clinical implementation across this group is the absence of standardized assays and of age- and sex-specific pediatric reference ranges.

### 3.3. Prognostic Biomarkers

Whereas the diagnostic markers reflect ongoing OS, a separate set of biomarkers captures the inflammatory consequences of redox imbalance and is therefore better suited for prognosis. The complex interactions among increased adipose tissue, IR and hepatic inflammation lead to hepatic fibrosis in the end stage [51,52,53], with OS fitting into this interplay by activating a multi-protein intracellular complex called the NLRP3 inflammasome, which releases interleukin 1β and interleukin 18, promoting hepatocyte injury and fibrosis [54] (Table 4). This proinflammatory cytokine phenotype correlates with local and systemic IR, potentiating the role of adipose tissue in inflammation.

Tumor necrosis factor alpha (TNF-α) is a proinflammatory cytokine, secreted primarily in visceral adipose tissue, that serves as both a product and amplifier of OS. Through complex mechanisms, it causes impaired insulin receptor signaling, enhances peripheral lipolysis, promotes hepatic lipogenesis, and activates stellate cells, contributing to fibrogenesis [52,55,56]. Elevated TNF-α is well documented in pediatric obesity and MetS, but it is not liver-specific and is influenced by systemic inflammation broadly [52,57]. Moreover, standardized reference ranges for children are lacking.

Interleukin 6 (IL-6) acts as a proinflammatory cytokine, induced by oxidative damage, interferes with normal insulin receptor activation and glucose metabolism, and has a lipolytic effect, leading to a consequent increase in free fatty acids in circulation. Increased serum concentrations have been found in patients with MASLD [51,52,55]. In the pediatric population, IL-6 has been linked to insulin resistance and MetS, though studies in children with MASLD remain scarce [58]. However, IL-6 is not specific to MASLD and is elevated across a wide range of inflammatory states, limiting its discriminatory value.

In contrast, IL-10, acting as an anti-inflammatory cytokine, has a protective role in patients with MASLD by limiting the secretion of the aforementioned proinflammatory cytokines, thus restraining inflammation [59]. In the same study, lower IL-10 levels correlated with a higher number of metabolic risk factors, including increased BMI, dyslipidemia, elevated blood pressure, and glucose intolerance, although pediatric reference ranges remain to be established [58].

A promising noninvasive prognostic biomarker for MASLD in pediatric populations is cytokeratin-18 (CK18), which is a significant intermediate filament protein in the liver, and caspase-cleaved CK18 fragments enter the bloodstream after apoptotic cell death. Studies showed that CK18 levels were significantly higher in children with MASH, and they even had excellent reliability in predicting the presence of MASH on liver biopsy [60,61].

**Table 4:** Prognostic biomarkers (inflammatory, apoptotic, and adipose-derived readouts downstream of oxidative stress).

Biomarker	Mechanism	Clinical Relevance	Limitations	Clinical Readiness
**TNF-α** [51,52,56]	Proinflammatory cytokine; impairs insulin receptor signaling, promotes peripheral lipolysis, hepatic lipogenesis, stellate-cell activation.	Elevated in obesity, MetS, and MASLD; driver of insulin resistance and steatosis; marker of disease progression.	Not liver-specific; no standardized pediatric reference ranges; reflects systemic inflammation.	**EMERGING** Research grade; no routine pediatric assay.
**IL-6** [51,52,62]	Proinflammatory cytokine induced by oxidative damage; impairs insulin signaling; lipolytic.	Elevated in prepubertal obese children and consistently elevated in MASLD.	Not specific; short half-life; elevated across many inflammatory states.	**EMERGING** Useful in research; measurement not standardized.
**IL-1β, IL-18** [54]	Released by NLRP3 inflammasome activation triggered by OS; promotes hepatocyte injury and fibrosis.	Link OS directly to hepatic inflammation and fibrosis progression.	No validated pediatric essays for routine use; evidence largely from animal and adult studies.	**RESEARCH ONLY** Animal/adult data only; no pediatric clinical assay.
**IL-10** [59]	Anti-inflammatory cytokine limiting TNF-alpha and IL-6 secretion.	Protective role in MetS and MASLD; proposed as a novel biomarker of metabolic risk.	No established pediatric reference ranges; inconsistent findings across studies.	**RESEARCH ONLY** Exploratory; no clinical implementation in children.
**CK-18** [60,61]	Caspase-cleaved cytokeratin-18 fragments—marker of hepatocyte apoptosis, the link between OS injury and steatohepatitis/fibrosis.	Best-validated noninvasive predictor of MASH and fibrosis in pediatric MASLD; combined panels (e.g., +leptin, +Ang-2) improve accuracy.	Assay/cutoff standardization still needed; overlaps other apoptotic states.	**EMERGING** Closest to clinically usable prognostic marker in children.
**Adiponectin, leptin, adiponectin/leptin ratio** [63,64]	Adipose-derived hormones reflect adipose dysfunction; adiponectin anti-inflammatory/insulin-sensitizing, leptin proinflammatory.	Track MASLD presence/severity in children (low adiponectin, high leptin); adiponectin/leptin ratio aids early detection.	Influenced by adiposity/puberty/sex; no pediatric cutoffs.	**EMERGING** Not yet ready for routine clinical use.

**Legend:** TNF-α—tumor necrosis factor alpha, MetS—metabolic syndrome, MASLD—metabolic dysfunction-associated fatty liver disease, IL—interleukin, CK-18—cytokeratin-18, OS—oxidative stress, MASH—metabolic dysfunction-associated steatohepatitis, EMERGING—Promising evidence based on pediatric-specific validation, standardized assays, or reference ranges is still lacking. RESEARCH ONLY—Evidence limited to adult/animal studies, single studies, or highly specialized platforms; not feasible for routine clinical use.

However, adipokines are important mediators of communication among adipose tissue, skeletal muscle, liver, and the vasculature in metabolic diseases [65,66]. Production of most adipokines is dysregulated in MetS and MASLD, with many of them playing important roles in the pathogenesis of inflammation and insulin resistance [65]. In this context, they have been identified as potential prognostic markers in MetS and MASLD.

Leptin is an adipocyte-derived hormone that modulates food intake and increases energy expenditure, facilitates glucose utilization, modulates lipid metabolism, improves insulin sensitivity, and modulates innate and adaptive immunity [62,66,67,68]. It is considered an anti-steatotic hormone, preventing lipid accumulation and promoting the mobilization of hepatic lipids. Elevated leptin levels are found in obese children, and correlate with the severity of hepatic steatosis [52,62,66,67].

Adiponectin, mostly expressed in adipose tissue, is inversely correlated with obesity. It regulates insulin sensitivity, inhibits liver neoglucogenesis, enhances fatty acid beta-oxidation, and decreases the production of inflammatory cytokines, protecting the liver from inflammation and fibrosis [66,67,68]. By reducing the production of TNF-α and IL-6, it counteracts their proinflammatory effects [67,69,70]. Reduced serum concentrations of adiponectin are associated with impaired fatty acid oxidation, increased lipogenesis and worsening of insulin resistance [52,67]. The leptin-to-adiponectin ratio is a more beneficial biomarker than either alone and can predict metabolic risk [71,72].

Taken together, the prognostic biomarkers discussed are not only markers of disease progression but also downstream readouts of the OS cascade. In patients with MASLD, they drive IR, promote hepatic steatosis and increase cardiometabolic risk [51,65,68]. Their prognostic utility in pediatric MASLD remains to be prospectively validated, as most available studies are cross-sectional.

### 3.4. Experimental Biomarkers

Beyond the established categories above, an emerging layer of investigation is reshaping the biomarker landscape. Metabolomics profiling is an emerging approach that focuses on the comprehensive analysis of dynamic changes in small molecules, or metabolites.

Although studies in the pediatric population are limited, available findings suggest characteristic changes primarily in amino acid and lipid metabolism [73]. Changes in lipoprotein levels, particularly the apolipoprotein B/apolipoprotein A-1 ratio, and glycoprotein acetyls have been identified as promising biomarkers in MetS [74] (Table 5). Amino acid metabolic disturbances influence MASLD pathogenesis. In particular, dysregulated branched-chain amino acids (BCAAs), aromatic amino acids (AAAs), and acylcarnitines were correlated with incomplete fatty acid oxidation, mitochondrial stress, and impaired insulin signaling [75,76]. Elevated lactate levels have been proposed as a potentially reliable early marker of IR in children. Longitudinal analyses have demonstrated elevated lactate concentrations that precede IR in non-obese children, suggesting deregulated hepatic glucose metabolism and mitochondrial impairment, making lactate levels an early precursor of IR. The same study also identified histidine levels as a potential early marker of IR in children [77]. Recent studies have revealed the pivotal role of gut microbiota dysbiosis and gut–liver axis dysfunction in MASLD patients. Disturbances in microbiota-derived secondary bile acids, short-chain fatty acids, and lipopolysaccharides are associated with inflammation and fibrosis in MASLD patients [78].

The multiomics approach integrates metabolomics with genomic (DNA), transcriptomic (ribonucleic acid—RNA), proteomic (proteins), and epigenomic analyses to provide an in-depth understanding of complex molecular interactions, disease mechanisms, and biomarkers. A large-scale multiomics study in children integrating multiple omics layers (transcriptomics, proteomics, metabolomics, microRNA expression and DNA methylation) identified and validated molecular signatures and distinct molecular clusters in childhood obesity. MicroRNAs (miR) are small RNAs which regulate gene expression at the posttranscriptional level and also engage in regulation, proliferation, differentiation, development, apoptosis and metabolism. They can be detectable in plasma, serum, urine and saliva. MiR-122 is a hepatic-specific miRNA which represents more than 70% of the whole hepatic miRNAome in humans. In adults, the liver and circulating miR-122 are related to severity of MASLD. In pediatric populations, levels of miR-122 are elevated in children with MASLD, and levels also rise with increasing severity of MASLD [79,80].

**Table 5:** Experimental biomarkers.

Biomarker	Mechanism	Clinical Relevance	Limitations	Clinical Readiness
**BCAAs, AAAs** [75,76,81]	Reflect mitochondrial stress and impaired insulin signaling.	Dysregulated in children with IR and metabolic disorders.	No standardized pediatric reference ranges; lacking studies in children; influenced by diet and physical activity.	**RESEARCH ONLY** Specialized platforms only; no clinical pediatric assay.
**Lactate** [77]	Reflects dysregulated hepatic glucose metabolism and mitochondrial impairment.	Elevated concentrations precede IR in non-obese children.	Findings from a single longitudinal study; clinical implementation not yet feasible.	**RESEARCH ONLY** Intriguing early marker; single study—replication needed.
**Histidine** [77]	Dysregulation is linked to early insulin resistance.	Proposed as an early marker of IR in children.	Findings from a single longitudinal study; clinical implementation not yet feasible.	**RESEARCH ONLY** Single study; requires independent replication.
**ApoB/ApoA-1 ratio** [73,74]	Reflects atherogenic lipoprotein balance.	Promising future biomarker for MetS.	Limited pediatric studies; requires longitudinal validation.	**EMERGING** Strong mechanistic rationale; validation in children needed.
**Glycoprotein acetyls** [73,74]	NMR-based inflammatory glycoprotein markers reflect systemic inflammatory status.	Promising biomarker for MetS.	Requires NMR platforms not routinely available; limited pediatric studies.	**RESEARCH ONLY** NMR platform required; not widely accessible.
**MiR** [79,80]	Liver-enriched/extracellular-vesicle miRNAs reflecting hepatocyte injury and fibrogenesis.	miR-122 shows high diagnostic accuracy for MASLD; panels track progression.	Preanalytical/normalization heterogeneity; few pediatric studies; platform-dependent.	**RESEARCH ONLY** Distinct emerging class; pulled from multiomics.
**Microbiota-derived markers (SCFAs, bile acids, LPS)** [78]	Gut–liver axis dysfunction.	Associated with inflammation and fibrosis in MASLD; represent potential therapeutic targets.	High inter-individual variability; no standardized clinical assays; limited pediatric data.	**RESEARCH ONLY** Therapeutic potential; no clinical assay available.
**Multiomics signatures** [81]	Integration of transcriptomics, proteomics, metabolomics, miRNA expression, and DNA methylation.	Validated molecular clusters of childhood obesity with metabolic dysfunction.	Extremely complex and costly; no standardized pediatric pipelines; very few longitudinal studies in children.	**RESEARCH ONLY** Research frontier; not feasible for routine clinical use.

**Legend:** BCAAs—branched-chain amino acids, AAAs—aromatic amino acids, IR—insulin resistance, MetS—metabolic syndrome, NMR—nuclear magnetic resonance, MASLD—metabolic dysfunction-associated fatty liver disease, SCFAs—short-chain fatty acids, LPS—lipopolysaccharide, miRNA—microRNA, DNA—deoxyribonucleic acid, EMERGING—Promising evidence based on pediatric-specific validation, standardized assays, or reference ranges is still lacking. RESEARCH ONLY—Evidence limited to adult/animal studies, single studies, or highly specialized platforms; not feasible for routine clinical use.

Metabolomic profiling further revealed a characteristic pattern of increased valine concentrations and low glycine levels, which was associated with IR and metabolic disorders [81]. Although integrative approaches appear to be the most feasible strategy for developing a clinically useful pediatric metabolic signature, their application remains experimental, with standardized longitudinal pediatric datasets currently scarce.

### 3.5. Selenium as a Modulator of Oxidative Stress

After reviewing the biomarker landscape, we now consider selenium as a potential modulator of OS, although pediatric evidence remains limited. Selenium (Se) is an essential micronutrient, incorporated into a family of selenoproteins, which makes an important contribution in critical antioxidant and redox-regulatory function [17]. Dietary selenium comes from a variety of foods, including meat, fish, eggs, dairy products, bread, etc. Selenomethionine is a principal form of it in nutrition, and in the liver it is bio-transformed to selenide, which serves as the precursor for selenoprotein synthesis.

The two most functionally significant selenoenzymes are GPx and thioredoxin reductase (TrxR) [82]. The principal forms of GPx are cytosolic, gastrointestinal-specific, plasma and phospholipid hydroperoxide, and their main purpose is prevention of oxidative damage via neutralization of hydrogen and organic peroxides, thereby limiting oxidative damage to cellular membranes and macromolecules (Table 6). TrxR manages apoptosis and proliferation of cells; has an important role in the elimination of different ROS; and modulates the activity of redox-sensitive transcription factors [83]. Together, these enzymes are key to selenium’s antioxidant role.

Beyond GPx and TrxR, three additional selenoproteins are relevant to MASLD. Selenoprotein P (SELENOP) shows the amount of Se bound to selenoproteins, indicates the liver levels of Se and takes part in extrahepatic Se transport and regulating lipid accumulation in the liver. As a result, adequate levels of SELENOP demonstrate that Se can perform all its functions in the body [84]. Selenoprotein S (SELENOS) is a significant selenoprotein, located in the endoplasmic reticulum, with an anti-inflammatory role. While in the pancreas and blood vessels, SELENOS has antioxidant protection and anti-ER stress effects, in the liver, adipose tissue, and skeletal muscle, SELENOS induces the start and development of IR [83]. Selenoprotein M (SELENOM) is a thioredoxin-like enzyme located in the endoplasmic reticulum and has an influence on redox control, fatty acid composition, stress responses and cancer cell proliferation. In liver diseases, selenoproteins have been demonstrated to reduce oxidative damage and dysregulation of mitochondrial dynamics in response to several types of stress. SELENOM loss may enhance obesity by influencing metabolic and endocrine functions [85].

In addition to these antioxidant roles, selenium participates in metabolic regulation by preventing overreactions which could lead to chronic inflammation and MASLD. Selenium possibly reduces metalloproteinase, TNF-α, IL-6 and other cytokines which take part in MASLD etiology, reducing hepatic inflammation and fibrosis [83].

The relationship between selenium exposure and metabolic health is complex and appears to be influenced by baseline selenium status, age, geographical region, and the biomarker used for its assessment. In children and adolescents with obesity, available studies have reported heterogeneous selenium concentrations and glutathione peroxidase activity, while pediatric reference ranges vary considerably across populations [17,86]. Adult evidence supports a U-shaped relationship: correcting selenium deficiency may restore antioxidant defenses, whereas additional supplementation in selenium-replete individuals may provide no further benefit and could increase the risk of insulin resistance (IR) and type 2 diabetes mellitus (T2DM) [87]. Evidence from adult trials suggests that prolonged selenium supplementation may modestly increase the risk of T2DM, particularly among individuals with higher baseline selenium status. This association was initially observed in a secondary analysis of a randomized controlled trial and was subsequently supported by a meta-analysis of five randomized trials [88,89]. Nevertheless, its generalizability remains limited because diabetes was a secondary outcome in the principal trial and the participants were predominantly older White adults.

These apparently opposing effects may reflect the dual influence of selenium on glucose metabolism and insulin signaling. Although adequate selenoprotein activity limits oxidative injury, physiological concentrations of reactive oxygen species also function as second messengers within the insulin-signaling pathway. Excessive selenium exposure may increase antioxidant selenoprotein expression and reduce the availability of these signaling molecules, thereby attenuating insulin action. In experimental models, glutathione peroxidase 1 overexpression has been associated with hyperinsulinemia and insulin resistance (IR), whereas increased liver-derived selenoprotein P may impair insulin signaling in the liver and skeletal muscle. Excessive selenium exposure may also disrupt pancreatic β-cell function and glucose and lipid metabolism [90]. Emerging pediatric evidence supports a nonlinear association, although it remains observational. In a cross-sectional study of 194 Mexican schoolchildren aged 6–10 years, 39.6% had serum selenium concentrations below 6.0 µg/dL and 5.2% had concentrations above 12.9 µg/dL. Compared with the reference range of 6.0–12.9 µg/dL, severe selenium deficiency (<4.5 µg/dL) was associated, after adjustment for nutritional status, with higher insulin concentrations (β = 1.9 µU/mL; 95% CI: 0.7–3.1), HOMA-IR (β = 0.4; 95% CI: 0.1–0.7), triglycerides (β = 17.0 mg/dL; 95% CI: 1.2–32.8), and triglyceride–glucose index (β = 0.2; 95% CI: 0.1–0.4). Nonlinear analyses additionally identified U-shaped associations of serum selenium with HOMA-IR (*p* = 0.016) and insulin concentrations (*p* = 0.044), suggesting that both insufficient and elevated selenium status may be metabolically unfavorable in children [91]. However, only 5.2% of participants had high selenium concentrations, the categorical associations between high selenium status and insulin or HOMA-IR were not statistically significant, and important potential confounders, including dietary selenium intake, pubertal development, physical activity, and inflammatory status, were not assessed. These mechanisms and pediatric observations may be relevant to metabolic dysfunction-associated steatotic liver disease (MASLD), in which IR is a central pathogenic feature, but the study did not specifically include children with MASLD. Consequently, the available evidence cannot establish causality, an optimal pediatric serum range, or a toxicity threshold. Until pediatric dose–response and long-term safety data become available, selenium supplementation should be individualized according to baseline selenium status, dietary intake, selenium formulation, and documented deficiency; routine high-dose supplementation for the prevention or treatment of pediatric MASLD cannot currently be recommended. Collectively, these findings reinforce the potentially narrow therapeutic window of selenium and the need to balance its antioxidant benefits against possible adverse metabolic effects.

**Table 6:** Selenium-related biomarkers.

Biomarker	Mechanism	Clinical Relevance	Limitations	Clinical Readiness
**Serum selenium status** [17,83,84,92,93,94]	Essential trace element; modulates inflammatory processes via SELENOP and GPx; supports antioxidant enzymes.	Deficiency reduces antioxidant enzymes and may lead to fatty liver; relevant to OS management.	Influenced by geographical soil Se content, dietary patterns, and bioavailability; no pediatric clinical guidelines on Se monitoring in MetS/MASLD.	**EMERGING** Measurable; no pediatric monitoring guidelines yet.
**SELENOP** [82,84,95]	Primary Se transport protein reflecting hepatic Se stores; regulates lipid accumulation.	Decreased in obese children; proposed as an early marker of MetS.	Limited pediatric data; reference ranges in children not established.	**RESEARCH ONLY** Limited pediatric evidence; not routinely measured.
**GPx** [17,83,92,96]	Selenoenzyme neutralizing peroxides and organic hydroperoxides via glutathione; GPx3 is the circulating, Se-status-responsive isoform.	Reduced in obese children with IR and in children with MASLD.	No standardized pediatric reference ranges; conflicting findings across studies.	**EMERGING** Measurable in serum/RBC; conflicting findings across studies.
**TrxR** [83,92]	Selenoenzyme regulating cellular apoptosis, proliferation, and ROS elimination.	Part of the Se-dependent antioxidant network in MetS and MASLD.	Limited pediatric data; measurement not routinely performed; no pediatric reference ranges.	**RESEARCH ONLY** Limited pediatric evidence; not routinely measured.
**SELENOS** [49,83,96]	Endoplasmic reticulum selenoprotein regulating ER stress and endoplasmic reticulum-associated degradation; hepatic deficiency promotes steatosis; paradoxically induces IR in metabolic tissues.	Dual role relevant to MASLD; hepatic deficiency exacerbates steatosis and ER stress in animal models; no direct pediatric data available.	Limited pediatric data; no clinical assay available.	**RESEARCH ONLY** Mechanistic role unclear in children; research only.
**SELENOM** [71]	Thioredoxin-like selenoprotein maintaining mitochondrial homeostasis via mitophagy regulation.	Reduces oxidative damage and mitochondrial dysregulation in liver diseases; loss associated with obesity and metabolic dysfunction.	Limited pediatric data; no clinical measurement available; mechanistic studies predominantly in animal models.	**RESEARCH ONLY** Animal models only; no pediatric clinical data.

**Legend:** SELENOP—selenoprotein P, GPx—glutathione peroxidase, TrxR—thioredoxin reductase, MASLD—metabolic dysfunction-associated fatty liver disease, SELENOS—selenoprotein S, SELENOM—selenoprotein M. EMERGING—Promising evidence based on pediatric-specific validation, standardized assays, or reference ranges is still lacking. RESEARCH ONLY—Evidence limited to adult/animal studies, single studies, or highly specialized platforms; not feasible for routine clinical use.

Evidence in children remains scarce and inconclusive. The pediatric data available are limited to a small number of cross-sectional studies, and findings are frequently contradictory. A cross-sectional study by Santos et al. in children aged 0–18 years showed that MASLD was accompanied by high body mass index and older age as well as reduced levels of GPx [97]. This finding is biologically consistent with the established mechanistic role of GPx. However, a notable paradox emerged in that erythrocyte GPx activity was elevated only in overweight children; in obese children, it was almost the same as in the control group. Plasma GPx levels in obese children with IR were reduced compared to lean and obese children without IR [92]. Several studies conducted on adults indicated that adults with lower blood Se levels had a higher percentage of liver damage [93,94].

The relationship between SELENOP and IR in obese children has been explored in a cross-sectional study by Elbarky et al. [95] which reported that reduced SELENOP levels increase IR. Although mechanistically likely, given the involvement of SELENOP in hepatic selenium transport and lipid regulation, the direction and clinical implications of this association in pediatric populations remain uncertain. The proposed role of SELENOP as an early marker of MASLD in obese children is biologically intriguing but should be regarded as preliminary, as it is currently supported by only one study and has not been confirmed in longitudinal cohorts, validated against pediatric reference ranges, or tested in intervention studies.

Overall, selenium and its related selenoproteins may play an important role in modulating oxidative stress in pediatric MASLD. Nevertheless, current evidence is still scarce and heterogeneous, making it insufficient to guide clinical practice. Well-designed prospective longitudinal studies are therefore needed to clarify how selenium status and selenoprotein dynamics relate to the development and progression of MASLD in pediatric populations.

**Figure 3:**
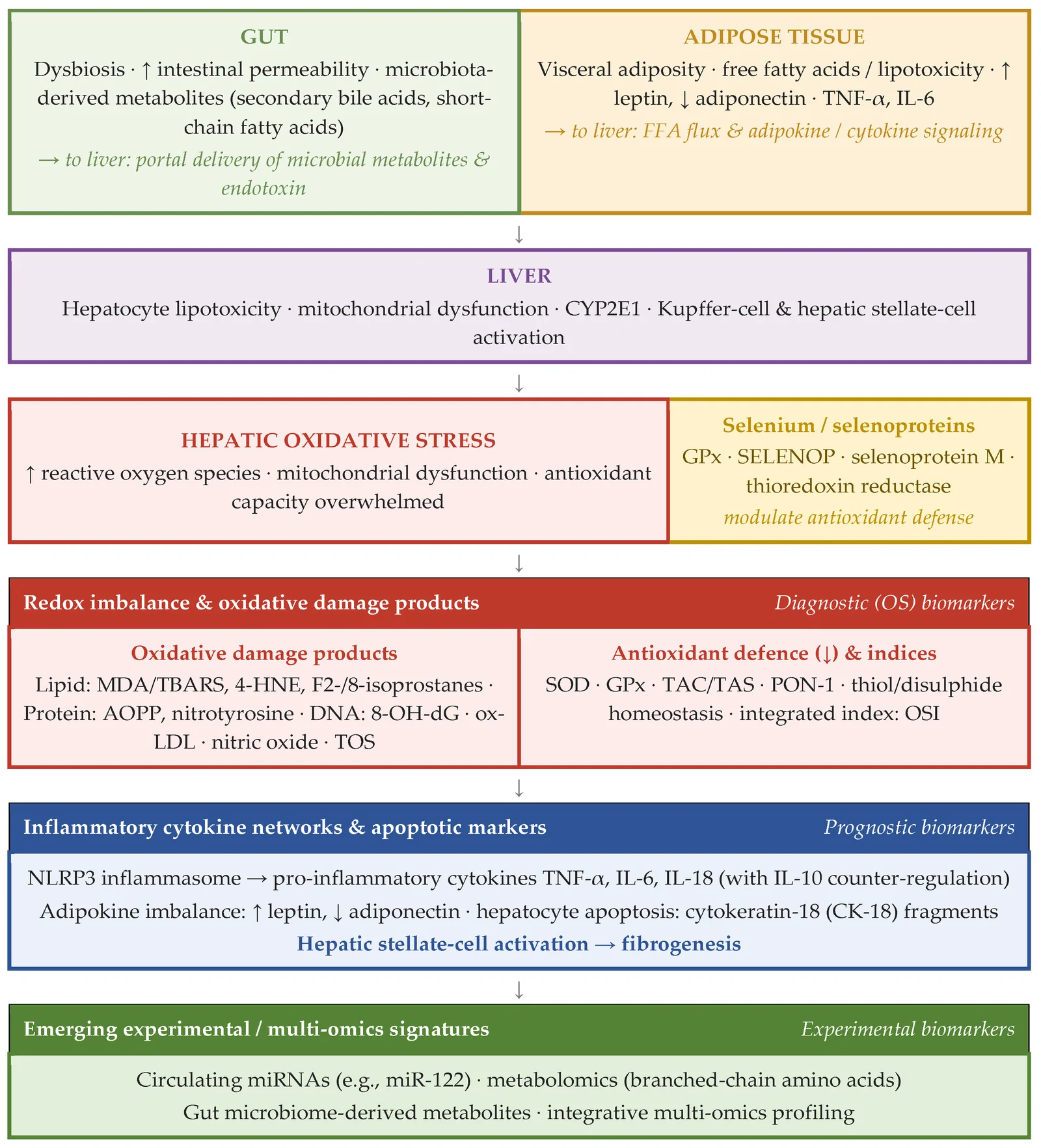
Integrated inter-organ crosstalk and the oxidative stress biomarker cascade in pediatric MASLD. ↑—increase, ↓—decrease.

The pathogenesis of pediatric MASLD involves a complex interplay between metabolic dysfunction, inter-organ communication, oxidative stress, inflammation, and tissue injury. An integrated overview of the proposed organ crosstalk, redox–metabolic pathways, and associated diagnostic, prognostic, and experimental biomarker networks is presented in Figure 3.

## 4. Clinical Implications and Future Research Directions

The biomarkers we reviewed here together illustrate the potential as well as current limitations in translation of OS research to everyday clinical practice in taking care of children with MASLD. From the clinical point of view, their basic value is for now more conceptual rather than operational: they confirmed that OS is a very early manifestation of pediatric MASLD which can be detected very early among most affected children, even before manifestation of liver injury. The redox imbalance is closely linked with IR, adipose dysfunction and low-grade inflammation, which are initiators of disease progression. This understanding supports a clinical focus on early detection in children who are at risk of MASLD, targeting the triggers of OS.

Several biomarkers are approaching possible clinical utility. Accessible and affordable indices derived from routine parameters, such as the ratio of uric acid and creatinine, along with integrated indices such as the oxidative stress index, are attractive because they can be automated and interpreted in healthcare settings. Among the prognostic markers, cytokeratin-18 and the leptin-to-adiponectin ratio show the clearest signals for noninvasive risk stratification. However, none of these are yet ready for routine clinical practice. One limitation is repeated in all biomarker categories: lack of standardization and, crucially, age- and sex-specific reference ranges for the pediatric population. Without these, interpretation on an individual basis remains unreliable and cutoff values cannot be defined for the purpose of clinical decision processes.

The next limitation is that a vast amount of the data are from cross-sectional studies, and most of the published associations so far indicate correlation rather than causality and still cannot be used for prognosis on the individual level. Future research should focus, through longitudinal studies in pediatric populations, on Se status and its impact on OS, because a vast amount of the data are from adult populations and still controversial.

Also, further studies should focus on standardization and confirmation of pediatric reference levels, without either of these biomarkers being used in clinical practice, and validation of composite indices and muti-omics signatures. The shared goal is helping clinicians develop further strategies for implementation of early and more accurate biomarkers of OS regarding MASLD.

## Data Availability

No new data were created or analyzed in this study. Data sharing is not applicable to this article.

## Abbreviations

The following abbreviations are used in this manuscript:

AOPPs	Advanced oxidation protein products
AAAs	Aromatic amino acids
BAP	Biological antioxidant potential
BCAAs	Branched-chain amino acids
BMI	Body mass index
CDAI	Composite Dietary Antioxidant Index
CAT	Catalase
CK-18	Cytokeratin-18
CYP2E1	Cytochrome P450 2E1
DNA	Deoxyribonucleic acid
dROMs	Reactive oxygen metabolites
GPx	Glutathione peroxidase
HOMA-IR	Homeostatic Model Assessment of Insulin Resistance
IL-10	Interleukin 10
IL-6	Interleukin 6
IR	Insulin resistance
MAFLD	Metabolic dysfunction-associated fatty liver disease
MASH	Metabolic dysfunction-associated steatohepatitis
MASLD	Metabolic dysfunction-associated steatotic liver disease
MDA	Malondialdehyde
MetS	Metabolic syndrome
NAFLD	Nonalcoholic fatty liver disease
NASH	Nonalcoholic steatohepatitis
NO	Nitric oxide
OBS	Oxidative balance score
Ox-LDL	Oxidized low-density lipoprotein
Ox-HDL	Oxidized high-density lipoprotein
PON1	Paraoxonase-1
RNS	Reactive nitrogen species
ROS	Reactive oxygen species
RNA	Ribonucleic acid
MiRNAs	MicroRNAs
NMR	Nuclear magnetic resonance
OS	Oxidative stress
OSI	Oxidative stress index
Se	Selenium
SELENOP	Selenoprotein P
SELENOS	Selenoprotein S
SELENOM	Selenoprotein M
SLD	Steatotic liver disease
SOD	Superoxide dismutase
suA/sCr ratio	Serum uric acid to creatinine ratio
TAC	Total antioxidant capacity
TBARS	Thiobarbituric acid reactive substances
TAS	Total antioxidant status
TOS	Total oxidant status
TrxR	Thioredoxin reductase
TNF-α	Tumor necrosis factor alpha
8-OH-dG	8-hydroxy-2′-deoxyguanosine
4HNE	4-Hydroxynonenal
